# Case report: Gross persistent rectal prolapse. A case treated without mesh using deep retrorectal dissection/suturing

**DOI:** 10.3389/fped.2022.900081

**Published:** 2022-08-18

**Authors:** Go Miyano, Shunsuke Yamada, Hiroshi Murakami, Geoffrey J. Lane, Atsuyuki Yamataka

**Affiliations:** Department of Pediatric General and Urogenital Surgery, Juntendo University School of Medicine, Tokyo, Japan

**Keywords:** rectal prolapse, laparoscopy, rectal fixation, barium enema, peritoneal reflection

## Abstract

A previously well 15-year-old male presented with a history of gross rectal prolapse (GRP) involving full-thickness rectal prolapse of increasing severity and incidence over 6 months that occurred with every bowel motion, varying from 10 to 40 cm. He denied constipation and passed a soft motion once daily, adeptly reducing his prolapsed rectum after each motion. This case illustrates technical challenges and planning for surgical intervention for optimal treatment in keeping with an FDA alert issued April, 2019 banning surgical mesh for pelvic organ prolapse. Preoperative fluoroscopic defecography confirmed rectal prolapse beginning with eversion of the anal verge identified on inspection. For surgery, general anesthesia was induced, he was placed in a Trendelenburg position, and four ports were inserted. The peritoneum was incised and blunt dissection used to expose the levator ani complex (LAC) taking care to prevent lateral nerve injury and preserve regional vascularity. Seven polypropylene sutures were used to fix the seromuscular posterior wall of the rectum to the median raphe of the LAC, the presacral fascia, and the periosteum of the sacral promontory. Operative time was 170 min. Postoperative recovery and progress were unremarkable. Currently, 5 years postoperatively, defecation is regular without recurrence of prolapse. For prolapse involving protrusion of the upper rectum without eversion of the anal verge, rectal fixation to the sacral promontory without further dissection beyond the peritoneal reflection is adequate, but when extensive prolapse is associated with eversion of the anal verge, more extensive blunt dissection from the peritoneal reflection to the LAC with multiple rectopexy sutures is valid for reducing risks for recurrence and eliminating mesh-related complications.

## Introduction

Various procedures have been described for the repair of gross rectal prolapse (GRP) with varying success. Successful surgical intervention and technical aspects of repairing GRP in an adolescent are presented.

## Case report

**FIGURE 1:**
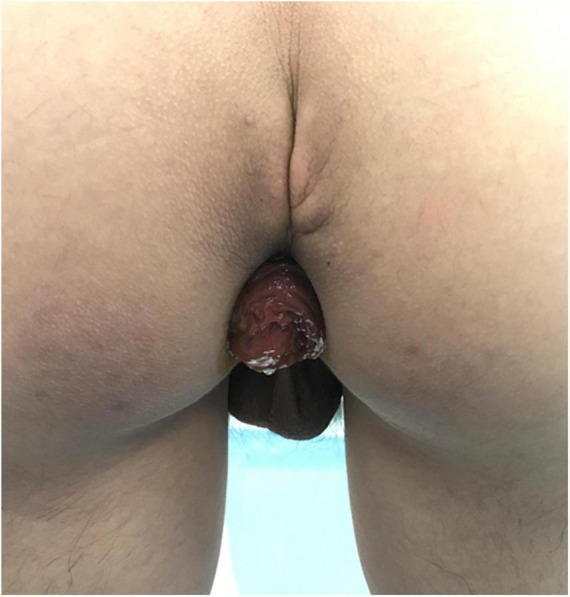
Gross rectal prolapse occurred after each bowel motion. The picture shows a small prolapse.

A 15-year-old Japanese male presented with a 6-month history of recurrent full-thickness GRP. He was an only child, otherwise well (height: 172 cm; weight: 61 kg) with no remarkable previous medical history; physical and mental status were normal; no specific family history or psychosocial issues were identified. He had no history of previous surgical interventions. Bowel function was regular (soft, well formed, once daily) without constipation. Prolapse occurred with every bowel motion without any obvious cause and varied from 10 to 40 cm (Figure 1). He adeptly reduced his prolapsed rectum after each motion and sought medical attention because of inconvenience.

**FIGURE 2:**
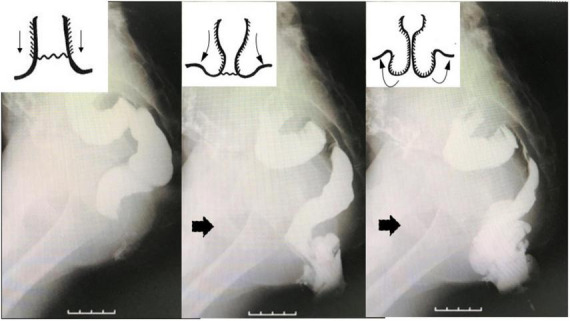
Fluoroscopic defecography identified extensive prolapse with eversion of the anal verge (arrows), not just protrusion of the upper rectum through the anus without eversion of the distal rectum.

On assessment, a trial of conservative management was recommended. Probiotics and laxatives were used daily to soften his stools to eliminate straining without any real improvement. After 6 months, fluoroscopic defecography was performed by filling the rectum with undiluted barium contrast agent and asking the patient to strain and expel the barium. Eversion of the anal verge was observed before more proximal rectum and sigmoid colon prolapsed on fluoroscopy (Figure 2). The extent of prolapse indicated that extensive repair was required rather than standard rectal fixation to the sacral promontory without further dissection beyond the peritoneal reflection when there is prolapse of the upper rectum without eversion of the anal verge. Because the patient denied constipation, structural integrity of the perineum was suspected as the cause for GRP. Levator ani muscle complex (LAC) integrity and anal sphincter function were considered crucial for success. Laparoscopic rectopexy with deep dissection beyond the peritoneal reflection and multiple sutures to the LAC parallel to the sacral promontory was planned.

**FIGURE 3:**
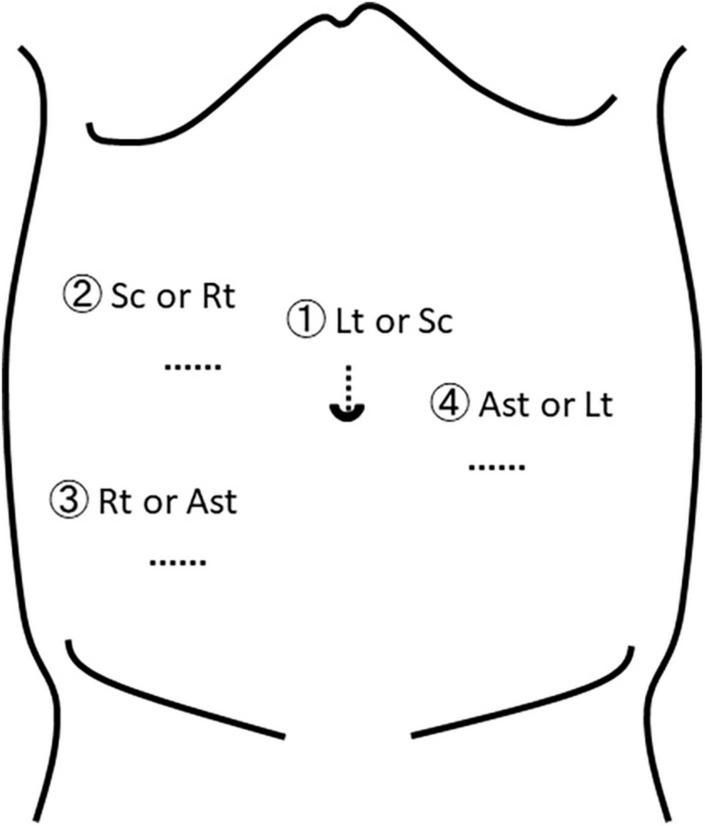
Trocar positions. The surgeon’s right and left hands should be located relatively close to each other to be able to reach the area of the levator ani complex. Sc, scope; Rt, surgeon’s right hand; Lt, surgeon’s left hand; Ast, assistant.

**FIGURE 4:**
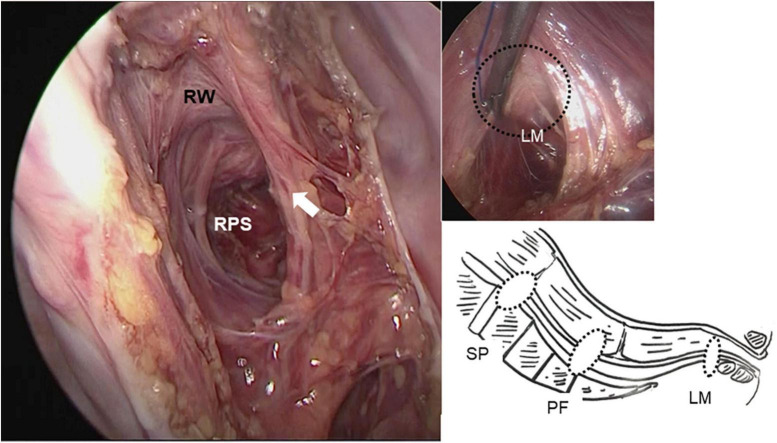
A retrorectal space was created from the peritoneal reflection to the sacral promontory extending deep into the pelvis around the levator ani complex using only blunt dissection, preventing lateral nerve injury and preserving lateral vascularity (white arrow). Rectopexy sutures were placed between the posterior wall of the rectum, the levator ani complex, the presacral fascia, and the sacral promontory (right-lower). RW, rectal wall; PRS, posterior rectal space; SP, sacral promontory; PF, presacral fascia; LM, levator ani muscle.

General anesthesia was induced with the patient supine and four ports (umbilical port for the scope, two lateral working ports, and a port for retraction of the rectum) were inserted, conventionally (Figure 3). The patient was then placed in a Trendelenburg position. On inspection, the rectosigmoid colon was found to be of normal caliber, without any caliber changes, but extremely floppy. Its peritoneum was incised on the right side of the rectum starting from the peritoneal reflection to the sacral promontory and blunt dissection was used to expose the LAC to prevent lateral nerve injury and preserve lateral vascularity (Figure 4). Seven polypropylene sutures (4-0 Prolene) were used to fix the seromuscular posterior wall of the rectum to the median raphe (levator plate) of the LAC, the presacral fascia, and the periosteum of the sacral promontory. All were tied extracorporeally (Figure 4). Operative time was 170 min with minimal blood loss. Postoperative recovery and progress was unremarkable without recurrence, and currently, 5 years postoperatively, he defecates a soft stool once daily without probiotics or laxatives. Postoperative management involved regular outpatient visits at increasingly longer intervals as his condition stabilized. He had no specific postoperative treatment and compliance was not an issue. From the patient’s perspective, defecation was stress-free.

Diagnosis, differential diagnosis, reasoning/indications for treatment and treatment goals, and prognosis were discussed with his caregivers according to CARE guidelines. Written informed consent was obtained from his caregivers for publication of his case report with images. A timeline figure was not included because diagnosis, treatment, and follow-up were straightforward.

## Discussion

The trend for repairing rectal prolapse in the literature is minimal retrorectal dissection without breaching the peritoneal reflection, preservation of the posterior rectal space and lateral rectal ligament, and localized rectopexy to the sacral promontory, a maneuver reported to effectively control recurrence to a rate of around 5% (1, 2). An alternative approach using mesh has an even lower recurrence rate and has been performed successfully in children (3), however, mesh-related complications, such as infection and erosion into the rectum or vagina (4) were of concern, and after the FDA ban of surgical mesh for pelvic organ prolapse in April, 2019, a mesh-free option was required. Thus, more extensive dissection from the peritoneal reflection to the LAC with multiple rectopexy sutures using non-absorbable material was chosen. In view of the density of neurovascular and venous plexuses on the pelvic surface of the sacrum, peanut gauze swabs were used for dissection to minimize local trauma during dissection and preserve lateral rectal ligaments. Electrocautery was not used for hemostasis to prevent compromising rectal blood supply and presacral innervation (2) that could hinder postoperative bowel function. As a result, dissection was mainly blunt rather than sharp, even though sharp dissection is standard in adult colorectal surgery. Blunt dissection was also considered likely to promote adhesion formation and be less invasive, especially if dissection happened to be inappropriate.

For preoperative planning, a defecography is mandatory despite concerns about radiation exposure (1). Magnetic resonance (MR) defecography is an option but dynamic defecography is probably easier to perform in children (5, 6) although MR defecography is better for imaging (6). Anorectal manometry is also useful. The exact nature of the prolapse must be confirmed categorically, especially specific features such as protrusion of the upper rectum from the anus, or eversion of the anal verge as well as the distal edge of the rectum (7). Barium enema alone is inadequate in GRP cases and does not allow eversion of the anal verge to be confirmed readily. During fluoroscopic defecography, the anal verge was observed to evert first, followed by prolapse of the proximal rectum while straining to expel the barium. If the rectum was fixed just to the peritoneal reflection, the distal rectum was considered to still be at risk for prolapsing; a possible cause for the 5% recurrence rate reported in the literature (1, 2) could be further reduced with GRP as in this case.

Deep retrorectal dissection was successful as a mesh-free option for treating GRP.

## Data availability statement

The original contributions presented in this study are included in the article/supplementary material, further inquiries can be directed to the corresponding author.

## Author contributions

GM treated the patient and prepared the manuscript. SY and HM treated the patient. GL revised the manuscript. AY supervised the entire drafting of the manuscript. All authors have read and approved the final manuscript and contributed to the article and approved the submitted version.
